# Effects of Fungicide Propiconazole on the Yeast-Like Symbiotes in Brown Planthopper (BPH, *Nilaparvata lugens* Stål) and Its Role in Controlling BPH Infestation

**DOI:** 10.3389/fphys.2019.00089

**Published:** 2019-02-11

**Authors:** Xuping Shentu, Xiaolong Wang, Yin Xiao, Xiaoping Yu

**Affiliations:** Zhejiang Provincial Key Laboratory of Biometrology and Inspection & Quarantine, College of Life Sciences, China Jiliang University, Hangzhou, China

**Keywords:** yeast-like symbiotes, *Nilaparvata lugens*, *Hypomyces chrysospermus*, microinjection, propiconazole

## Abstract

Yeast-like symbiotes (YLS), harbored in the abdomen fat-body cells of the rice brown planthopper (BPH), *Nilaparvata lugens* Stål (Hemiptera: Delphacidae), are vital to the growth and reproduction of their host. It is feasible to manipulate BPH infestation on rice by inhibiting YLS using fungicide. In this study, the fungicide propiconazole was injected into the hemolymph of BPH thorax via microinjection to investigate its effect on YLS, especially the dominant species, *Hypomyces chrysospermus*, and their host BPH. Propiconazole markedly reduced the total number of YLS and *H. chrysospermus* in BPH hemolymph and fat body, thereby leading to an obvious higher mortality and lower fecundity of BPH than the negative control (PBS, phosphate buffer solution). After microinjecting propiconazole, the survival rate of BPH nymphs at the 5th instar was significantly lower than that obtained after PBS treatment. Eight days after propiconazole microinjection, the BPH survival rate dropped to 40%, only half of BPH survival rate treated with PBS microinjection. For female adults (1-day-old), there were significant differences in the survival rates between BPHs treated with propiconazole and those treated with PBS at days 5–8. The fecundity of BPH decreased significantly by microinjecting propiconazole and averaged only 229 eggs per female, which was 20% less than that of the negative control. Furthermore, we reared BPH on the susceptible variety TN1 sprayed with propiconazole to prove the feasibility manipulating field occurrence of BPH by inhibiting YLS using fungicides. The number of YLS and *H. chrysospermus* in BPH obviously declined. Subsequently, the survival rate and fecundity of BPH significantly decreased after feeding on rice treated with propiconazole. Meanwhile, the propiconazole residue was detected in the hemolymph and gut of BPH by HPLC analysis within 1 day of feeding. Inhibiting YLS using fungicides was a novel and effective way to control BPH infestation.

## Introduction

The brown planthopper (BPH), *Nilaparvata lugens* Stål (Hemiptera: Delphacidae), is one of the most destructive monophagous insect pests of rice in Asia (Park et al., 2008). The insects suck nutrients from the phloem of rice plants and transmit plant viruses such as ragged stunt and grassy stunt viruses (Ge et al., 2011; Piyaphongkul et al., 2012; Wan et al., 2013). High BPH populations can destroy rice plants and cause hopper burn in a short period of time (Yang et al., 2002). Up to now, the control of BPH has predominantly relied on the use of synthetic chemicals (Puinean et al., 2010; Wan et al., 2013). However, due to the injudicious use of chemical insecticides, BPH has evolved a high level of resistance to major varieties of insecticides, including organophosphates, carbamates, pyrethroids, neonicotinoids, insect growth regulators, and phenylpyrazoles (Wu et al., 2018). In recent years, BPH outbreaks have occurred more frequently in China and other Asian countries, thereby causing a serious yield loss of rice (Wang et al., 2008; Wu et al., 2018). Thus, the global importance of rice, which supplies approximately 20% of the world’s calorific intake, drives research on the development of BPH control methods.

Yeast-like symbiotes (YLS), harbored in the fat body cells of BPH abdomen, are dominant obligatory symbionts, although several bacterial symbionts have been reported (Tang et al., 2010). BPH establishes an intimate symbiotic relationship with YLS and provides a small habitat for YLS (Chaves et al., 2009). In turn, YLS has vital physiological and trophic functions in the growth and reproduction of their host BPH and provides complementary functions to their host such as essential amino acid synthesis, nitrogen storage and recycling, steroid synthesis, and vitamin supply (Xue et al., 2014).

In order to clearly elucidate the close relationship between YLS and its host BPH, many experiments have been conducted on the taxonomy and diversity of YLS populations. Kagayama et al. (1993) isolated seven morphologically different YLS strains from the eggs of BPH. In our previous studies, we used 18S rDNA and internal transcribed spacer (ITS)–5.8S rDNA sequences analysis, *Cryp*-Like and *Pichia*-Like YLS symbiotes were identified (Dong et al., 2011). Furthermore, several fungal species, namely, *Hypomyces chrysospermus* (usually called *Noda*), *Pichia guilliermondii*, *Candida* sp., *Saccharomycetales* sp., and *Debaryomyces hansenii*, were detected by nested PCR-denaturing Gradient Gel Electrophoresis (DGGE) technology (Hou et al., 2013). Therefore, many species of YLS exists in BPH, according to these reports. Up to now, the species and amounts of YLS in the fat body of BPH keep unknown. *H. chrysospermus* is the dominant species of YLS in BPH (Noda et al., 1995; Cheng and Hou, 1996).

The YLS in the fat body of BPH is transmitted to the next generation by transovarial infection (Cheng and Hou, 1996, 2001). The transmission process is as follows: the symbiotes in mycetocytes move out of the syncytium, which is formed from a layer of fat body cells, by exocytosis and release into BPH’s hemocoel in BPH females. Then, the free YLS in hemolymph approach the ovarioles near the pedicel and are enclosed by follicle cells. They enter the follicle cells around the primary oocyte by endocytosis at epithelial plug of the ovariole. The YLS aggregate at the posterior end of the mature egg after entering and finally form a symbiote ball (Cheng and Hou, 2001). Furthermore, the entry of YLS into BPH oocyte was triggered by oocyte vitellogenesis, as shown in our previous study (Nan et al., 2016). Whether or not the growth and fecundity of BPH are affected by the number of total YLS and *H. chrysospermus* in the transovarial process is unclear. In this paper, the fungicide propiconazole was injected into the hemolymph of BPH thorax using microinjection technology to observe its effects on the YLS and their host BPH. Furthermore, we reared BPH on the susceptible variety TN1 sprayed with propiconazole to prove the feasibility of manipulating BPH infestation by inhibiting YLS using fungicides. Inhibiting YLS using fungicides was a novel and effective way to control BPH infestation.

## Materials and Methods

### Insect Mass Rearing and Rice Culture

Brown planthopper population used in the experiments were originally collected from rice fields in Hangzhou (E120°12, N30°16), China. Successive generations were maintained on the susceptible rice variety TN1 in a climatic chamber under constant conditions of 26 ± 1°C, 70–80% relative humidity and a 16 h light/8 h dark photoperiod. The TN1 seedlings were cultured in 14 cm diameter plastic pots and used for BPH mass rearing at the tillering stage (height: 14–16 cm).

### Microinjection of Fungicide Into Hemolymph of BPH Thorax

The fungicide 50% propiconazole ME was provided from Qingdao Hengyuanxiang Chemical Co., Ltd., Propiconazole was diluted with 0.01 mol/L phosphate buffer solution (PBS) to 0.17 ng/nL. The final concentration for microinjection was determined by the preliminary gradient experiments. Thirty BPH nymphs in the 5th instar or 1-day-old female adults were used for microinjection for each treatment with five replications. The BPH individuals were anesthetized using CO_2_ for 30 s and fixed on 4% agarose plate with their abdomen facing up. Propiconazole at 17 ng (100 nL) was injected into each BPH thorax using the FemtoJet 4i microinjection device (Eppendorf, Germany), and the injection sites were located on the conjunction between prothorax and mesothorax (Lu et al., 2015). The treatments with 0.01 mol/L PBS and without microinjection were used as the negative and the blank controls, respectively. BPH samples were collected at day 1, day 2, and day 4 after injection to investigate the number of YLS and *H. chrysospermus*. The mortality and fecundity of BPH were investigated and calculated correspondingly.

### Effects of Propiconazole With Foliar Spraying on Rice Plants

Foliar spraying with propiconazole was carried out at the rice tillering stage using a mini-sprayer. Propiconazole was used at the recommended concentration (0.5 mg/mL) in the field trial. Three stems of sprayed rice plants were placed in a test tube (2.5 cm in diameter and 30 cm in height) filled with 20 ml rice nutrient solution (Wilkinson and Ishikawa, 2001; Shentu et al., 2016). Thirty fifth-instar nymphs or 1-day-old female adults were released into the test tube, which was covered with two pieces of gauze. These were then kept in a constant temperature room at 26 ± 1°C with 16 h light/8 h dark photoperiod. Five replications were performed in each treatment. The treatment sprayed with water only was used as the negative control. The mortality and fecundity of BPH were calculated. The number of YLS and *H. chrysospermus* in the BPH body was investigated at day 1, day 2, and day 4 after BPH release.

### Quantification of Total YLS

Brown planthopper samples were sterilized by immersion in 75% ethanol for 3 min and then washed quickly with 0.01 mol/L PBS for 90 s. The fat bodies in the BPH abdomen and the hemolymph in the BPH thorax were collected by dissection and homogenized in 0.01 mol/L ice-cold PBS at pH 7.4 Percoll (Pharmacia, Sweden), respectively (Shentu et al., 2016). The total number of YLS in the fat body and the hemolymph were counted on a hemocytometer under a binocular microscope (400 ×). Each sample was observed in triplicate.

### Quantification of *H. chrysospermus* by Quantitative Real-Time PCR (qPCR)

Total DNA of YLS in the hemolymph and fat body was extracted using a Yeast DNA Mini Kit (Tiangen Biotech Co., Ltd., Beijing, China). The DNA concentration was measured using a spectrophotometer (Nanodrop). To estimate the abundance of *H. chrysospermus*, the copy number of the 18S rDNA (Genbank: AF267233.1) fragment was measured by qPCR (Applied Biosystems) using a FastStart Universal SYBR Green Master(ROX) (Roche Biotechnology Co., Ltd.), and the two primers used for qPCR amplification were F: 5′-CGTAGGAGAGCAGCAAAC-3′ and R: 5′-CGATGCCAGAGCCAAGA G-3′. The *actin* (Genbank: KU365929.1) was used as the reference gene. The primers were F:5′-GATGAGGCGCAGTCAAAGAG-3′ and R:5′-GTCATCTTCTCACGGTTGG C-3′. The primers were designed by Primer Premier 5.0 with DNA sequence and cDNA sequence. The resulting PCR products were cloned into a pMD18-T vector (TaKaRa Biotechnology (Dalian) Co., Ltd.). The inserted gene fragments were sequenced and were proven to correspond to a part of the target gene. The qPCR was performed in a 20 μL total reaction volume containing 10 μL of FastStart Universal SYBR Green Master(ROX), 2 μL of template DNA, 0.8 μL of forward primer (10 μM), 0.8 μL of reverse primer (10 μM), and 6.4 μL of ddH_2_O. The qPCR reactions were pre-denatured at 95°C for 10 min, followed by 40 cycles of 95°C for 15 s and 60°C for 1 min. Each DNA template was analyzed in triplicate. The acceptable qPCR standard curve (0.9 ≤*E* ≤ 1.0, *R*^2^ ≥ 0.99) for each gene was optimized by altering the annealing temperature and time. The normalized fold changes of the target gene DNA copy number were expressed as 2^-ΔΔCT^ (Schmittgen and Livak, 2008).

### Analysis of Propiconazole Residue in BPH

Propiconazole standard sample was purchased from Aladdin–Holdings Group. Fifteen BPH individuals in group were dissected, and their hemolymph and gut were collected and homogenized in 0.01 mol/L PBS at pH 7.4 Percoll (Pharmacia, Sweden), respectively (Shentu et al., 2016). Then, the PBS extraction solution was subjected to centrifugation (12,000 r/min, 5 min) to obtain the supernatant. The 500 μL supernatant was filtrated with 0.22 μm filter membrane. The residue of propiconazole was analyzed using a Waters e2695 HPLC (2998 PDA Detector, Waters Workstation, United States) (Mu et al., 2005). The mobile phase was methanol and water (85:15, v/v). The detection wavelength was 225 nm, and the flow speed was 1.0 mL/min. The column (RP-C18 column) temperature was maintained at 30°C (250 mm × 4.6 mm, 5 μm, XBridgeTM, Waters, United States).

### Data Analysis

Values were expressed as mean ± SE. All data were analyzed with SPSS, version 24.0. Comparisons of the means were conducted based on Least Significant Difference (LSD) test following a one-way analysis of variance (ANOVA). Differences between means was deemed significant and highly significant when *P* < 0.05 and *P* < 0.01, respectively.

## Results

### Effect of Propiconazole on the Number of YLS in BPH

**FIGURE 1:**
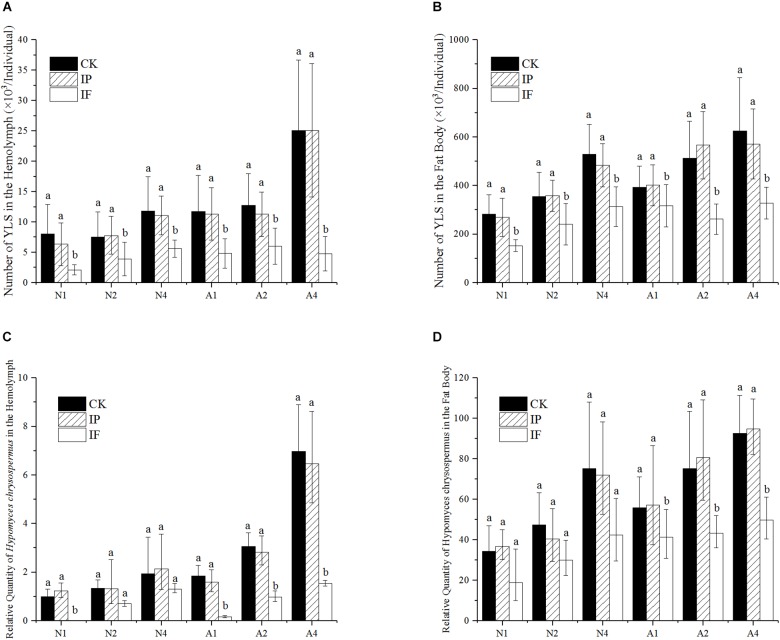
Effect of propiconazole on the number of YLS in BPH. **(A)** Effect of propiconazole on the total number of YLS in the hemolymph. **(B)** Effect of propiconazole on the total number of YLS in the fat body. **(C)** Effect of propiconazole on *H. chrysospermus* in the hemolymph. **(D)** Effect of propiconazole on *H. chrysospermus* in the fat body. CK, untreated control; IP, PBS microinjection; IF, propiconazole microinjection; N, BPH nymphs in the 5th instar; A, 1-day-old female adults; 1, 2, and 4: 1 day, 2 day, and 4 day after microinjection, respectively. One-way ANOVA with LSD test was used to compare all individual treatments with one another. Significant differences were indicated by different letters at *P* < 0.05. The quantification of *H. chrysospermus* 18S rDNA copy number in the different treatments was analyzed by 2^-ΔΔCt^ method. 1 day after untreated BPH nymphs in the 5th instar were used as control.

Effect of propiconazole on the YLS in hemolymph and fat body of BPH was indicated in Figure 1A–D. The total number of all YLS in BPH generally increased with the growth of host BPH (Figure 1A,B). One day after propiconazole microinjection, the total number of YLS in the hemolymph of nymph decreased as low as 33.6% of that in the negative control (PBS treatment). At days 2 and 4 after microinjection, the numbers of YLS in the treatment with propiconazole significantly declined to 48.7 and 50.6% as compared with that in the negative control, respectively (*P* < 0.05). Similar results were also obtained from the treatment of BPH female adults. The total number of YLS in the hemolymph of female adults obviously declined when treated with propiconazole. Especially at day 4 after microinjection, the number of the YLS dropped to 18.9 % of that in the negative control (PBS treatment) (Figure 1A).

The total number of YLS in the BPH fat body after propiconazole microinjection was significantly lower than that of untreated or PBS-treated controls (*P* < 0.05). For the 5th instar nymph and 1-day-old female adults, the growth and reproduction of YLS was effectively suppressed at day 1 (a drop of 43.3 and 21.2%), day 2 (a drop of 32.7 and 53.8%), and day 4 (a drop of 35.2 and 42.6%) after propiconazole microinjection as compared with PBS microinjection, respectively (Figure 1B).

The number of *H. chrysospermus* was analyzed after microinjection (Figure 1C,D). In the 5th instar BPH nymph, the number of *H. chrysospermus* increased with BPH growth. However, the number of *H. chrysospermus* in the BPH hemolymph treated with propiconazole was significantly decreased compared with that of the PBS-control (*P* < 0.05). On 1-day-old female adults, the number of *H. chrysospermus* had rapid declined at day 1 (a decline of 90%), day 2 (a decline of 65%), and day 4 (a decline of 76%) after propiconazole microinjection as compared with PBS microinjection (Figure 1C). Meanwhile, a big decline in the number of *H. chrysospermus* was observed after propiconazole microinjection in BPH fat body (Figure 1D).

### Effect of Propiconazole on the Survival Rate of BPH

**FIGURE 2:**
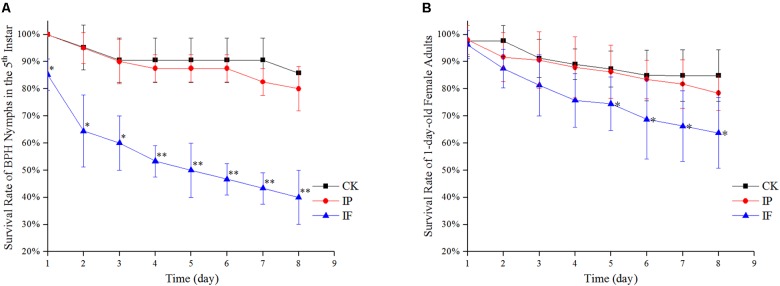
Effect of propiconazole on the survival rate of BPH. **(A)** Effect of propiconazole on the survival rate of BPH nymphs in the 5th instar. **(B)** Effect of propiconazole on the survival of 1-old-day female adults. CK, untreated control; IP, PBS microinjection; IF, propiconazole microinjection. One-way ANOVA with LSD test was used to compare the treatments between propiconazole microinjection and PBS microinjection. Significant differences were indicated by “^∗^” (*P* < 0.05) and “^∗∗^” (*P* < 0.01).

The survival rates of BPH by hemolymph microinjection were indicated in Figure 2A,B. During days 1 and 3 after microinjection, there was a significant difference in the survival rates of the 5th instar nymph between BPH treated with propiconazole and PBS (*P* < 0.05) (Figure 2A). Furthermore, from days 4 to 8 after propiconazole microinjection, the survival rate of 5th instar nymph was highly significantly lower than that of the control treated with PBS (*P* < 0.01). The BPH survival rate was 40% after microinjection at day 8, which was lower by half of the BPH survival rate after treatment with PBS microinjection (Figure 2A). During days 5 and 8, the survival rates of 1-day-old female adults treated with propiconazole were significantly different from those treated with PBS (*P* < 0.05) (Figure 2B).

### Effect of Propiconazole on the Fecundity of BPH

**FIGURE 3:**
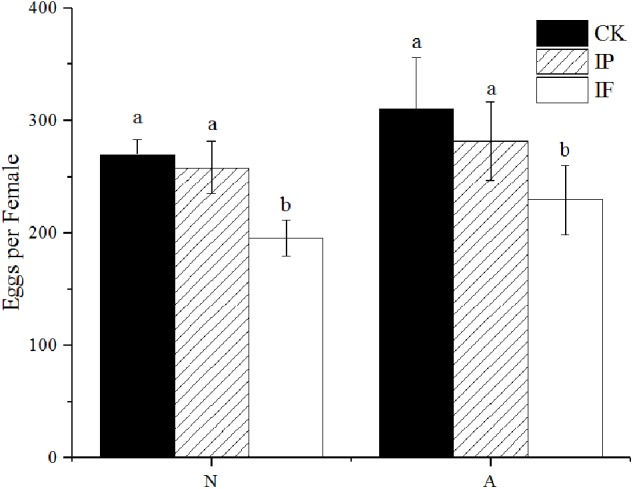
Effect of propiconazole on fecundity of BPH. CK, untreated control; IP, PBS microinjection; IF, propiconazole microinjection; N, BPH nymphs in the 5th instar; A, 1-day-old female adults. One-way ANOVA with LSD test was used to compare all individual treatments to each other. Significant differences were indicated by different letters at *P* < 0.05.

The fecundity of BPH females significantly decreased after propiconazole microinjection (*P* < 0.05) (Figure 3). Female adults (1-day-old) laid 229 eggs per female after propiconazole microinjection, which was 20% lower than the control treated with PBS. For BPH treated in the 5th instar nymph, the oviposition of BPH treated with propiconazole was 195 eggs per female, which was only 75% of the PBS treatment.

### Effect of Propiconazole on the Number of YLS in BPH by Foliar Spraying

**FIGURE 4:**
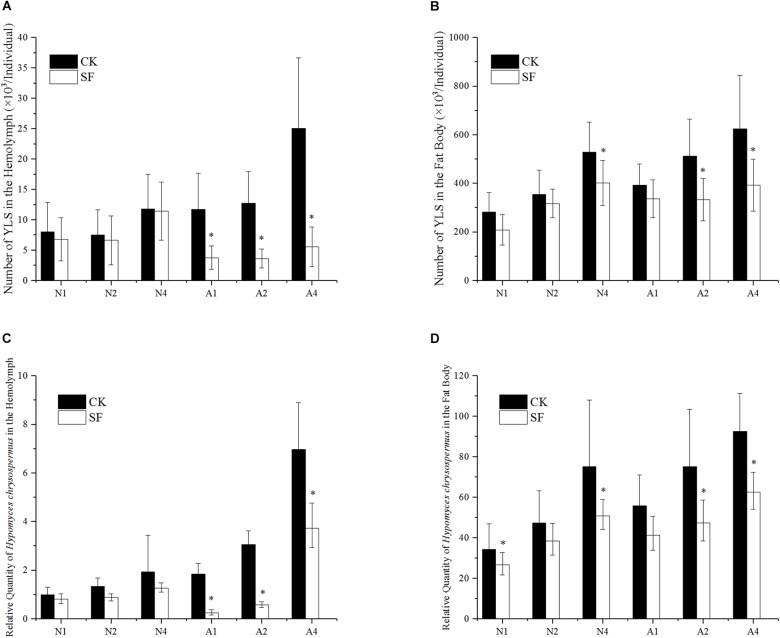
Effect of propiconazole on the number of YLS by foliar spraying. **(A)** Effect of foliar sprayed propiconazole on the total number of YLS in the hemolymph. **(B)** Effect of foliar sprayed propiconazole on the total number of YLS in the fat body. **(C)** Effect of foliar sprayed propiconazole on *H. chrysospermus* in the hemolymph. **(D)** Effect of foliar sprayed propiconazole on *H. chrysospermus* in the fat body. CK, water control; SF, foliar spraying with propiconazole; N, BPH nymphs in the 5th instar; A, 1-day-old female adults; 1, 2, and 4, 1 day, 2 day, and 4 day after foliar spraying with propiconazole. One-way ANOVA with LSD test was used to compare all individual treatments with one another. Significant differences were indicated by “^∗^” (*P* < 0.05). The quantification of *H. chrysospermus* 18S rDNA copy number in the different treatments was analyzed by 2^-ΔΔCt^ method. 1 day after water treatment of BPH nymphs in the 5th instar was used as control.

The effect of fungicide on YLS after foliar spraying was shown in Figure 4A,B. From day 1 to day 3 after spraying, the number of YLS in the BPH hemolymph in 5th instar nymph was similar to that of water-treated BPH. However, the numbers of YLS in 1-day-old female adults significantly declined to 32, 29, and 22% of the negative control at day 1, day 2, and day 4 after foliar spraying, respectively (Figure 4A).

In the fat body of the 5th instar nymph, the number of YLS reduced significantly at day 1 and day 4 after foliar spraying with propiconazole compared with the water-treated control (*P* < 0.05). Meanwhile the growth and reproduction of YLS in 1-day-old female adults was also significantly inhibited at day 2 and day 4 after foliar spraying with propiconazole (*P* < 0.05) (Figure 4B).

The number of the dominant YLS species, *H. chrysospermus*, decreased after foliar spraying with propiconazole in the 5th instar nymphs and 1-day-old female adults (Figure 4C,D). Particularly, the number of *H. chrysospermus* in 1-day-old female adults significantly decreased at day 1 (a drop of 86%), day 2 (a drop of 81%), and day 4 (a drop of 81%) after foliar spraying with propiconazole compared with the negative control (*P* < 0.05) (Figure 4C). In the BPH fat body, an obvious reduction in the number of *H. chrysospermus* at day 2 and day 4 was observed after foliar spraying with propiconazole (Figure 4D).

### Effects of Propiconazole on the Survival and Fecundity of BPH by Foliar Spraying

**FIGURE 5:**
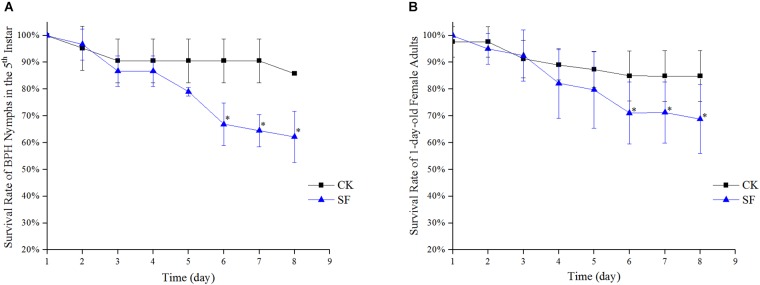
Effect of propiconazole on the survival rate of BPH by foliar spraying. **(A)** Effect of foliar sprayed propiconazole on the survival rate of BPH nymphs in the 5th instar. **(B)** Effect of foliar sprayed propiconazole on the survival of 1-old-day female adults. CK, water control; SF, foliar spraying with propiconazole; One-way ANOVA with LSD test was used to compare all individual treatments to each other. Significant differences were indicated by “^∗^” (*P* < 0.05).

From day 1 to day 5 after foliar spraying with propiconazole, there was no significant difference between the treatment and negative control in both the 5th instar nymph and 1-day-old female adults (*P* < 0.05) (Figure 5A,B). Furthermore, from day 6 to day 8 after foliar spraying, the survival rates of the 5th nymph and 1-day-old female adults of BPH showed significant differences between propiconazole and water treatments (*P* < 0.05).

**FIGURE 6:**
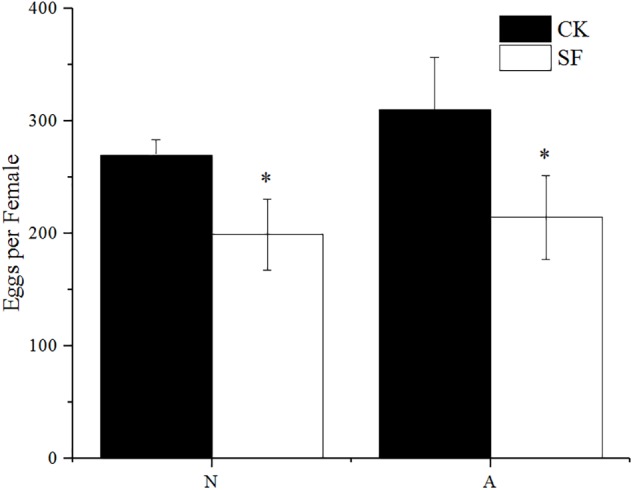
Effect of foliar sprayed propiconazole on fecundity of BPH. CK, water control; SF, foliar spraying with propiconazole; N, BPH nymphs in the 5th instar; A, 1-day-old female adults. One-way ANOVA with LSD test was used to compare all individual treatments to each other. Significant differences were indicated by “^∗^” (*P* < 0.05).

The fecundity of BPH significantly decreased after foliar spraying with propiconazole (Figure 6). The BPH treated with fungicide at the 5th nymph and 1-day-old female adults laid 199 and 214 eggs per female, respectively, which were 27 and 31% drops compared with the water-treated BPH.

### Propiconazole Residue in the Hemolymph and Gut of BPH After Treatments

**FIGURE 7:**
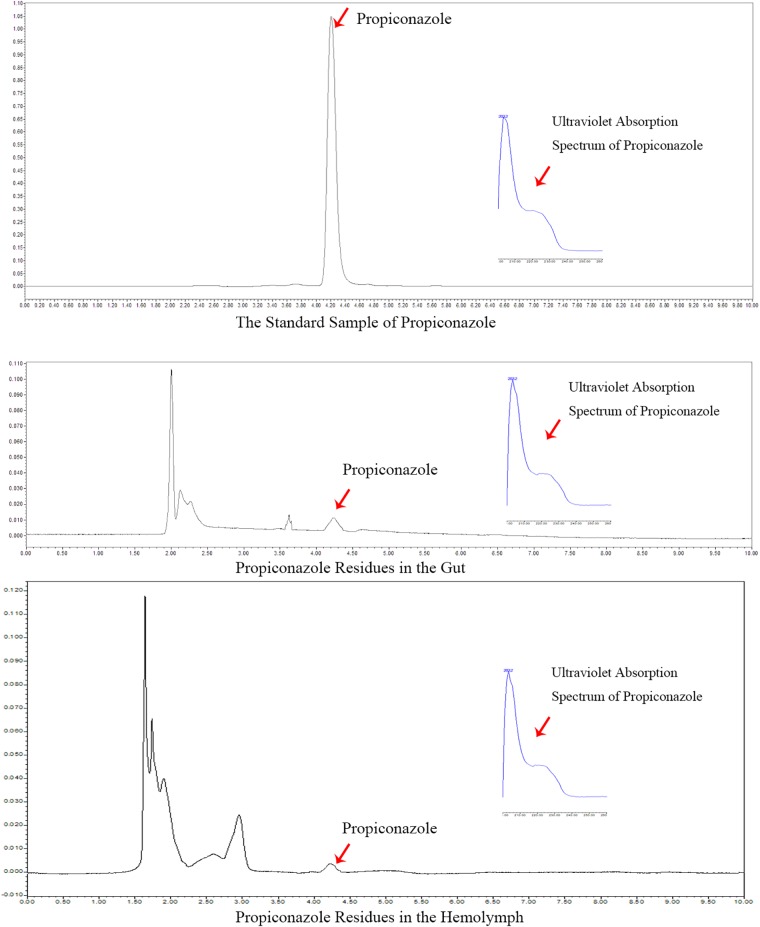
HPLC analysis of propiconazole in the hemolymph of BPH.

In the foliar spraying test, the residue of propiconazole in the hemolymph and gut was detected at day 1 after BPH release and the result was shown in Figure 7. According to the retention time and ultraviolet absorption spectrum of propiconazole by HPLC, there were propiconazole residues in the hemolymph and gut of BPH, respectively. The concentration of propiconazole residue in the gut was higher than in the hemolymph.

## Discussion

Yeast-like symbiotes harbored in the fat body of BPH abdomen plays a vital role in the growth and reproduction to their host BPH. If the YLS in BPH significantly decreased, then BPH would not survive (Chang et al., 2011; Lee and Hou, 1987). Based on the intimate relationship between BPH and YLS, we tried to manipulate BPH population by inhibiting YLS using fungicides as additive of imidacloprid. Results showed the satisfactory effect of some fungicides on the abundance of YLS and the mortality of BPH in our previous study (Shentu et al., 2016). In order to clearly clarify the importance of YLS on their host BPH, the fungicide propiconazole was injected into the hemolymph of BPH to investigate its role on YLS and their host BPH. Propiconazole could cause a marked reduction of the total number of YLS in BPH’s hemolymph and fat body, thereby resulting in a significantly higher mortality and lower fecundity as compared with the PBS treatment. Hence, the decrease of YLS number in BPH not only caused BPH mortality but also reduced fecundity of BPH.

In our previous study, there was no significant difference in the survival rate of nymphs at 1 day after treatment with water spray and fungicide spray (Shentu et al., 2016). It may take a certain time for the fungicide to enter the hemolymph of BPH. However, at 1 day after propiconazole microinjection, the survival rate of the 5th instar BPH nymphs was significantly lower than that of the negative control (PBS treatment). Undoubtedly the inhibitory effect of propiconazole microinjection into the hemolymph of BPH on YLS was stronger than that of foliar fungicide sprays on rice plants. Furthermore, this study directly showed that fungicide treatment led to the decrease of YLS, thereby resulting in the high mortality and low fecundity of BPH. For newly emerged females, there was significant difference in the BPH survival rates between propiconazole treatment and PBS-treated control until day 5 to day 8 after microinjection. BPH females may have better tolerance to YLS loss because they have complete physiological function, including more nutritional reserves and numerous mycetocytes in the fat body. Thus, the same dose of fungicide had less inhibition effect on the YLS in BPH adults than that on BPH nymphs.

Yeast-like symbiotes in the fat body of BPH is transmitted into the next generation by transovarial infection (Cheng and Hou, 1996, 2001). By microinjecting propiconazole into the hemolymph of BPH, the transovarial process of YLS was obviously affected. Thus, the fecundity-related pathways were possibly affected by YLS decrease. As a result, the BPH fecundity was significantly affected. The fungicide could influence the growth of BPH and the reproduction as well.

Using microinjection of fungicide into the hemolymph of BPH is not practical in BPH control. Effects of propiconazole on the YLS and BPH by foliar spraying showed that the number of YLS and the survival rate of BPH obviously decreased in the rice field. Furthermore, the propiconazole residue was detected in the hemolymph and gut of BPH using HPLC analysis. Thus, the fungicide was assimilated by BPH feeding and then entered the hemolymph. The fungicide led to the decrease of YLS in BPH’s hemolymph and resulted in the death of BPH and lower fecundity. However, the fungicide may inhibit the microorganisms in BPH intestines. The interaction between BPH and microorganisms in BPH’s intestines needs further study.

According to previous reports, *H. chrysospermus* is the dominant species of YLS in BPH (Cheng and Hou, 1996, 2001). Hence, effects of propiconazole on *H. chrysospermus* in BPH using microinjection or foliar spraying were also studied. Similar results were found, the number of *H. chrysospermus* was significantly reduced in both nymphs and adults after treatments. Meanwhile we tried to detect the number variation of other YLS species, such as *Pichia guilliermondii*, *Candida* sp., *Saccharomycetales* sp., and *D. hansenii*, by qPCR technology, but the results were less well developed. One of the possible reasons was the number of these symbiotes was much less than that of the dominant species *H. chrysospermus*. Thus, it is difficult to quantify the number of these symbiotes by qPCR. DNA extraction efficiency and PCR reaction condition are required in future research to test the effect of other YLS species on the BPH.

We tested the direct influence of propiconazole on BPH. However, the elimination of intracellular symbiotes was difficult and rarely successful. BPH died if YLS was completely inhibited. We did not obtain the YLS-free BPH as the control in hemocoel microinjection and foliar spraying tests using fungicide. Thus, it is difficult to confirm the direct influence of propiconazole on BPH *per se*. In our previous study, the number of YLS in the BPH treated with imidacloprid was not reduced significantly (Shentu et al., 2016). In contrast, the mixture of fungicides with imidacloprid substantially reduced the number of YLS and subsequently caused high mortality of BPH. This finding indicated that some fungicides could significantly enhance the suppressive effect of the insecticide against the BPH population after the inhibition of YLS in BPH. Our research presents a new way to manipulate BPH occurrence through inhibition of YLS.

## Conclusion

Our study demonstrated that the fungicide caused the decrease of YLS, thereby resulting in the higher mortality and lower fecundity of BPH. Hence, inhibiting YLS using fungicide is a novel and effective way to control BPH infestation. To our knowledge, this is first report on the effect of fungicide microinjection into the hemolymph on YLS and BPH.

## Author Contributions

XS and XY conceived and designed the experiments, and wrote the manuscript. XW and YX performed the experiments. XS and XW analyzed the data.

## Conflict of Interest Statement

The authors declare that the research was conducted in the absence of any commercial or financial relationships that could be construed as a potential conflict of interest.
